# USP25 attenuates the immunosuppressive tumor microenvironment via the deubiquitination of TAB2 in head and neck squamous cell carcinoma

**DOI:** 10.1038/s41420-025-02883-1

**Published:** 2025-12-01

**Authors:** Xingchen Li, Yidi Jia, Runfang Zhang, Xu Zheng, Chuang Li, Weike Ma, Yang Han, Chen Zheng, Yanqing Li, Qianqian Shi, Hu Hei, Songtao Zhang, Jianwu Qin

**Affiliations:** 1 Department of Thyroid Head and Neck Surgery, Affiliated Cancer Hospital of Zhengzhou University, Henan Cancer Hospital, Zhengzhou, Henan China; 2 Institute of Cancer Research, Henan Academy of Innovations in Medical Science, Zhengzhou, Henan China; 3 Department of Gynecology and Obstetrics, The Second Affiliated Hospital of Zhengzhou University, Zhengzhou, Henan China; 4 Department of Pathology, The Second Hospital & Clinical Medical School Lanzhou University, Lanzhou, Gansu China; 5 Department of Laboratory Medicine, The Third Affiliated Hospital of Zhengzhou University, Zhengzhou, Henan China

**Keywords:** Cancer microenvironment, Cancer immunotherapy

## Abstract

The role of deubiquitinating enzymes in the tumor immune microenvironment (TIME) remains understudied. Here, we sought to identify the mechanisms of USP25 modulation in the TIME of head and neck squamous cell carcinoma (HNSCC). Bioinformatics analysis was performed to screen differentially expressed novel deubiquitinases (DUBs) in HNSCC. The importance of USP25 in clinical practice was assessed in the TCGA dataset and tissue microarrays. Single-cell RNA-sequencing was applied to profile the TIME. The function of USP25 was determined through loss-of-function assays. Reduced expression of USP25 was associated with the malignant progression of HNSCC and further indicated poor prognosis. USP25 protein levels were positively correlated with CD8^+^ T-cell infiltration in HNSCC tissue cohorts, suggesting its role in modulating the TIME. Concordantly, this study revealed a reduction in myeloid-derived suppressor cells (MDSCs), concomitant with increased numbers of cytotoxic T cells in tumors with high USP25 expression. Mechanistically, we revealed that USP25 binds to TAB2, removes K63-linked ubiquitination chains, and subsequently activates MAPK signaling and the secretion of IL-6, which increases MDSCs migration. Increased MSDCs in turn antagonized functional CD8^+^ T cells in the TIME. Importantly, overexpression of USP25 increased anti-PD1 therapeutic efficacy in HNSCC in vivo. These results underscore the critical role and mechanism of USP25 in modulating the TIME in HNSCC, suggesting its potential as a therapeutic target in immune checkpoint blockade therapy.

## Introduction

Head and neck cancer is the third most prevalent cancer in developing nations, and the sixth most prevalent cancer worldwide [1]. Among all the neoplasms of the head and neck region, approximately 90% are classified as head and neck squamous cell carcinoma (HNSCC) [2]. HNSCC originates from various sites in the head and neck area, such as the larynx, oropharynx, hypopharynx, and oral cavity [3], and its etiologic factors include smoking, betel quid chewing, and alcohol consumption; moreover, human papillomavirus infection is a risk factor for some types of HNSCC [4]. Most cases are in the advanced clinical stage at the time of diagnosis, characterized by low survival rates and a high incidence of regional lymph nodes or distant metastases [5]. More recently, immune checkpoint blockade (ICB) has demonstrated therapeutic benefits for patients with advanced HNSCC; however, only a minority of patients have positive responses to ICB, which is partly due to conditions of the suppressive tumor immune microenvironment (TIME) [6, 7]. Therefore, understanding the molecular mechanisms of immune evasion in HNSCC and identifying treatment strategies to improve the TIME and enhance the response to immunotherapy are highly important.

In recent years, the suppressive TIME, characterized by the presence of inhibitory cells that prevent T cells from entering tumor islets, has attracted wide spread attention in tumor research. The complex interaction between tumor cells and the TIME is profoundly interconnected with the initiation, invasion, and therapeutic response of a diverse array of tumors, such as HNSCC [8]. Indeed, the TIME represents an intricate ecological system shaped by the dynamic interplay between malignant cells and immune cells, making tumors a heterogeneous disease that is difficult to treat [9]. Tumor cells often orchestrate an immunosuppressive TIME through interactions with regulatory T cells (Tregs), tumor-associated macrophages (TAMs), and myeloid-derived suppressor cells (MDSCs), thereby dampening the antitumor immune response [10]. For example, Bao et al. demonstrated that tumor-derived YTHDF1 impairs antitumor immunity via the m6A-p65-CXCL1/CXCR2 axis to promote colorectal cancer [11]. Recently, combination therapies aimed at converting “cold” tumors into “hot” tumors have emerged as promising therapeutic modalities for immunotherapy-resistant patients [12]. Liao et al. reported that IRF2 overexpression or CXCR2 inhibition could increase the sensitivity of colorectal cancer cells to anti-PD-1 therapy [13]. Additionally, Li et al. revealed that combination therapy with a PI3K inhibitor significantly enhanced the therapeutic efficacy of ICB therapy in patients with breast cancer [14]. Despite these discoveries, their clinical translation remains limited, underscoring the need for a deeper understanding of the regulatory mechanisms underlying the conversion of “cold” tumors into “hot” tumors to improve the efficacy of ICB therapy.

Protein ubiquitination is an important posttranslational modification for ubiquitin-related protein degradation via the ubiquitin-proteasome system, and plays a pivotal role in protein degradation and cellular homeostasis [15]. Ubiquitination is a reversible process that is regulated by both E3 ligases and deubiquitinating enzymes (DUBs), which attach or remove ubiquitin from target proteins [16]. DUBs remove ubiquitination modifications from substrates, modify ubiquitin chains, and process ubiquitin precursors. DUBs such as USP19 [17], PSMD14 [18], USP47 [19], and UCHL1 [20] have been intensively studied in cancer. Notably, aberrant expression of ubiquitin specific peptidase 25 (USP25) has been reported to be associated with poor prognosis in various cancer types and evidence suggests that it promotes tumor growth, metastasis, and the chemotherapeutic response [21–23]. However, the role of USP25 in the TIME is largely unclear.

In this study, we reported that USP25 expression was dramatically decreased in HNSCC and reported a direct correlation between decreased USP25 expression, lymph node metastasis, and decreased survival in HNSCC patients. Utilizing a 4-NQO-induced HNSCC model, we revealed a decrease in USP25 expression during HNSCC malignant progression. Furthermore, scRNA-seq analysis revealed that USP25-low patients had a suppressive immune-infiltrating TIME characterized by reduced T-cell infiltration and increased MDSC accumulation. Mechanistically, the depletion of USP25 in HNSCC cells could induce the migration of MDSCs and inhibit the infiltration of T cells. Furthermore, we elucidated that USP25 binds to TAB2 and decreases the extent of ubiquitination through K63-linked chains, thus enhancing MAPK signaling activation and interleukin-6 (IL-6) secretion in HNSCC cells. Moreover, overexpression of USP25 enhanced the efficacy of anti-PD-1 therapy in HNSCC in vivo, highlighting its potential as a therapeutic target. These findings reveal a novel role and regulatory mechanism of USP25 in the TIME, providing a rationale for targeting USP25 in immunotherapy for advanced HNSCC patients.

## Results

### Reduced USP25 expression predicts poor prognosis in HNSCC patients

**Fig. 1:**
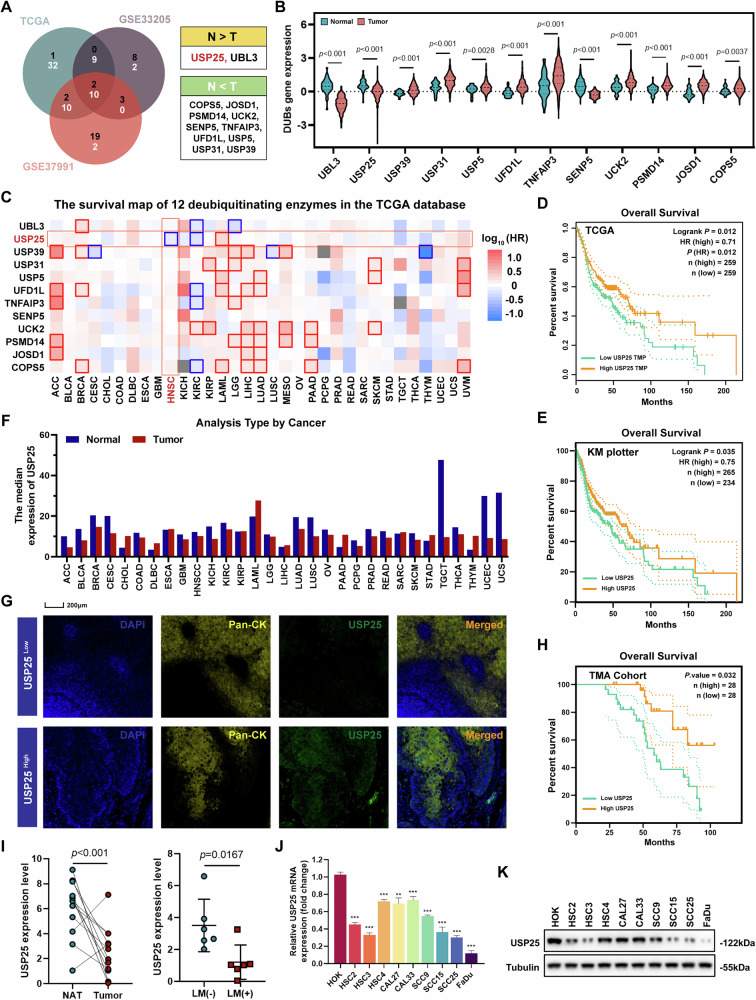
Reduced USP25 expression predicts poor prognosis in HNSCC patients. **A** Venn diagrams showing the differentially expressed DUBs in HNSCC. N normal tissue, T tumor tissue. **B** Violin plots showing the expression levels of 12 DUBs in the TCGA HNSCC database. **C** Survival map of 12 DUBs in pan-cancer generated by GEPIA2. **D** Kaplan–Meier survival curve analysis revealed that reduced expression of USP25 indicated an unfavorable prognosis in the TCGA HNSCC dataset. **E** Survival curve of HNSCC patients grouped according to USP25 expression status (high or low) according to overall survival. Dataset: Kaplan–Meier plotter. **F** Analysis of the TCGA database revealed that the level of USP25 was decreased in a broad spectrum of human cancers, that included HNSCC. **G** mIHC images showing the distribution of USP25 protein expression in HNSCC tissues from the TMA cohort (*n* = 56). **H** The DFS probability of HNSCC patients with high vs. low expression of USP25. **I** Left panel: qPCR analysis of the expression of USP25 in HNSCC tissues (*n* = 12) and NATs (*n* = 12). NAT normal adjacent tissue. T, tumor tissue. Right panel: qPCR analysis of the expression of USP25 in HNSCC tissues with (*n* = 6) or without (*n* = 6) lymphatic metastasis (LM). **J** USP25 expression was measured at the mRNA and protein levels (**K**) in the HOK cell line and a panel of HNSCC cell lines. Data represent the mean ± SD, *P < 0.05, **P < 0.01, ***P < 0.001.

To explore the potential regulatory mechanisms of DUBs in HNSCC pathogenesis, we conducted a comprehensive bioinformatics analysis leveraging multiomics datasets. We analyzed the expression profiles of 95 DUBs in HNSCC tissues and normal tissues from the GSE33205 and GSE37991 GEO datasets, and the TCGA database. Through an integrated Venn diagram analysis (Fig. 1A, B), we identified 10 significantly upregulated DUBs (COPS5, JOSD1, PSMD14, UCK2, SENP5, TNFAIP3, UFD1L, USP5, USP31, and USP39) and 2 significantly downregulated DUBs (USP25, and UBL3) in HNSCC compared with nonmalignant tissues. To elucidate the clinical implications of these DUBs, we used GEPIA2 to evaluate their prognostic significance across all the cancer cohorts. Notably, reduced USP25 expression emerged as the only DUB associated with inferior overall survival rates in HNSCC patients (Fig. 1C). Furthermore, Kaplan–Meier analysis based on the TCGA data revealed that HNSCC patients with lower USP25 expression had worse clinical outcomes (Fig. 1D), which was reconfirmed by the Kaplan–Meier plotter database (Fig. 1E), suggesting a potential prognostic role of USP25 in HNSCC. In addition, bioinformatics analysis revealed a consistent pattern of USP25 downregulation in multiple cancers (Fig. 1F), suggesting a potential role in the malignant progression of cancers. Moreover, we monitored the expression of USP25 in HNSCC patients using mIHC staining (Fig. 1G). The Kaplan–Meier analysis on the basis of the TMA HNSCC cohort also corroborated these results (Fig. 1H). Total RNA was extracted from fresh normal adjacent tissues (NATs) and tumor tissues. qRT-PCR revealed a significant reduction in USP25 expression in HNSCC tissue samples compared with that in NATs. Furthermore, modest upregulation of USP25 expression was observed in lymph node-negative (LN^−^) tumors, whereas in lymph node-positive (LN^+^) tumors, USP25 expression was markedly downregulated (Fig. 1I). Finally, qPCR (Fig. 1J) and western blotting (Fig. 1K) were performed to measure USP25 mRNA and protein levels in head and neck cell lines, including normal human oral keratinocyte (HOK), and HNSCC cell lines, and the results indicated that USP25 expression was decreased in all HNSCC cell lines compared with that in HOK cells. Taken together, these results revealed that USP25 expression is downregulated in HNSCC tissues and cell lines and that the loss of USP25 expression is potentially correlated with poor prognosis.

### USP25 regulates the malignant progression of HNSCC in vivo

**Fig. 2:**
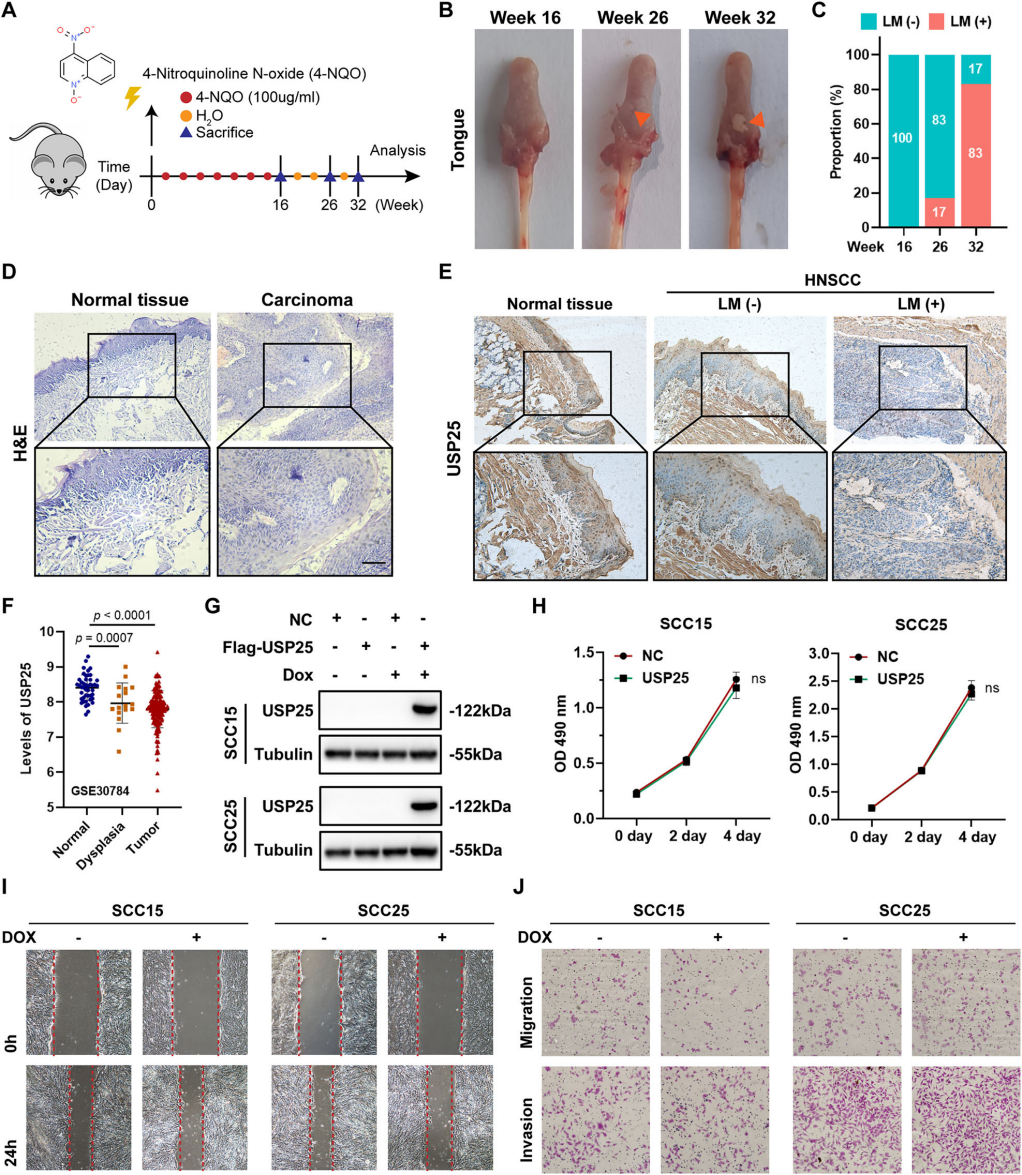
USP25 regulates malignant progression in HNSCC in vivo. **A** A model of oral carcinogenesis was established in mice by 4-NQO drinking water. **B** Representative images of tongue specimens from mice collected at weeks 16, 26, and 32. **C** The statistical graph shows the percentage of patients with lymph node metastasis. **D** HE staining of tumor tissues and normal tissues. **E** The results of IHC staining show the levels of USP25 in the indicated groups. **F** The expression of USP25 in normal tissue, dysplasia tissue, and tumor tissue are shown. **G** Western blot analysis of the SCC15 and SCC25 cell lines. **H** CCK8 assays for the SCC15 and SCC25 cell lines. **I** Wound-healing assays for HNSCC cells overexpressing USP25. **J** Transwell assays for HNSCC cells with USP25 overexpression. Data represent the mean ± SD, *P < 0.05, **P < 0.01, ***P < 0.001.

We established a 4-nitroquinoline N-oxide (4-NQO)-induced murine HNSCC model (Fig. 2A), and tongue specimens were collected at weeks 16, 26, and 32 (Fig. 2B). As shown in Fig. 2C, as the tumor size increased, the lymph node metastasis rate gradually increased. The hematoxylin-eosin (HE) staining results for both tumor and normal tissues are presented in Fig. 2D. IHC results showed significant downregulation of USP25 expression in carcinoma tissue compared with normal tissue. In particular, the tumor tissues with lymph node metastasis presented the lowest expression levels of USP25, suggesting that USP25 may be involved in HNSCC progression and is associated with metastasis (Fig. 2E). Interestingly, analysis of a GEO dataset (GSE30784) confirmed the findings derived from the aforementioned analysis and demonstrated that the levels of USP25 gradually decreased from normal tissues through dysplasia lesions, to carcinoma (Fig. 2F), suggesting that USP25 downregulation is an early and sustained event in malignant transformation. To further evaluate this, we knocked-down USP25 in a normal HOK cell line (Fig. S1A). Our results demonstrate that USP25 depletion in normal cells does not significantly affect proliferation or migration (Fig. S1B, C), supporting the notion that USP25 downregulation primarily exerts its functional effect in the context of HNSCC progression rather than in normal cellular physiology. To assess the direct effect of aberrant USP25 expression on tumor cells, we established cell lines via lentiviral transduction to stably express doxycycline-induced Flag-USP25 and investigated the effects of USP25 overexpression on the proliferation, migration, and invasion of tumor cells in vitro (Fig. 2G). The results of the CCK8 and clonogenic assays (Fig. S1D) for evaluating cell proliferation revealed no difference between the Dox-induced USP25 HNSCC cells and control cells (Fig. 2H). Furthermore, the results of the wound healing (Fig. 2I) and Transwell (Fig. 2J) assays revealed comparable migration patterns and invasive abilities in both groups of cells. Taken together, these results suggest that although USP25 significantly influences the malignant progression of HNSCC cells in vivo, it does not affect the biological function of HNSCC cells in vitro.

### Low expression of USP25 is correlated with an immunosuppressive TIME in HNSCC

**Fig. 3:**
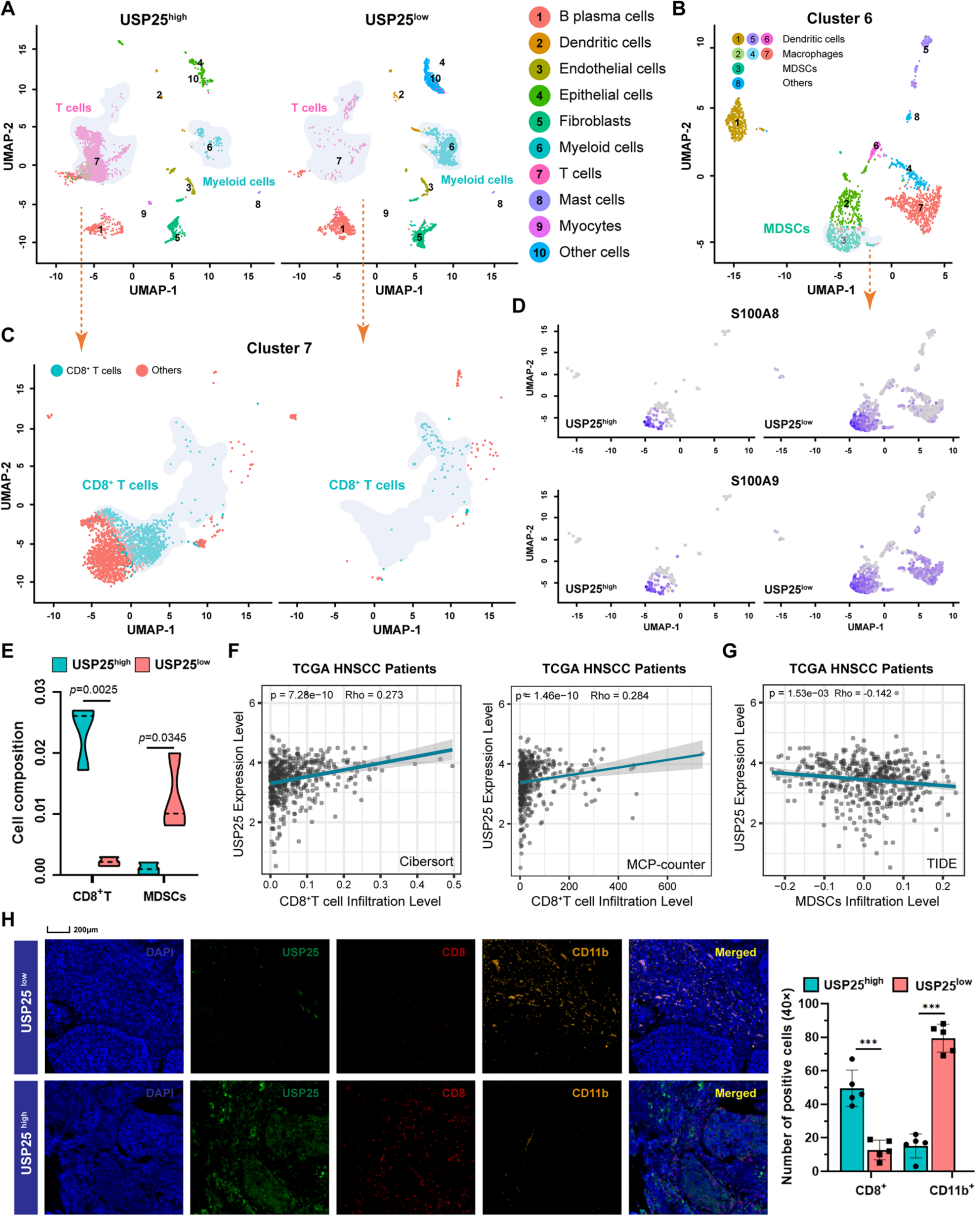
Low expression of USP25 is correlated with an immunosuppressive TIME in HNSCC. **A** t-SNE analysis showing the components of immune cells in HNSCC tumors on the basis of single-cell data from the GEO database GSE181919. **B** Subset analysis of myeloid cell and T-cell clusters (**C**) shown in t-SNE projection regions. **D** Feature plots of characteristic markers of all cell types showing expression levels with low expression in gray to high expression in violet. **E** Violin plots displaying the proportions of CD8^+^ T cells and MDSCs in the indicated groups. **F** Analysis of the correlation between USP25 expression and CD8^+^ T-cell or MDSC infiltration (**G**) infiltration in HNSCC patients in the TIMER public database. **H** mIHC staining for USP25, CD11b^+^ (MDSCs), and CD8^+^ (T cells) in tumor tissues from the HNSCC TMA cohort, and the summarized results. Data represent the mean ± SD, *P < 0.05, **P < 0.01, ***P < 0.001.

Tumor progression is profoundly influenced by the TIME, and an immunosuppressive TIME has been demonstrated to facilitate tumor growth and metastasis [24]. As important components of the immunosuppressive TIME, immunosuppressive cells can promote malignant progression by impeding the rejection of tumor cells by tumor-reactive T cells. To investigate the immunomodulatory effects of USP25 on the TIME, we analyzed the TIME with scRNA-seq (GSE181919) from the GEO database (Fig. 3A). The dataset includes the results of scRNA-seq profiling of normal, leukoplakia, HNSCC, and metastasized HNSCC tissues. The focus of the study design was on the microenvironment associated with HNSCC progression, making it a suitable resource to validate USP25 downregulation across tumor stages. On the basis of on the clustering results of the single-cell data and annotation information, UMAP dimensionality reduction was used to visualize the expression pattern of single cells. We subsequently executed unsupervised clustering of myeloid cells and T cells. A total of 8 clusters emerged within the myeloid lineage, including three clusters for dendritic cells, three clusters for macrophages, one for MDSCs, and one for other cells (Fig. 3B). We further categorized T cells into CD8^+^ T cells and other cell subsets and detected a simultaneous decrease in the proportion of T cells in the USP25^low^ group compared with that in the USP25^high^ group (Fig. 3C). Functional markers of MDSCs, such as S100A8 and S100A9, were examined, and these genes were enriched mainly in MDSC clusters, especially in clusters from tumors with low USP25 expression (Fig. 3D). Therefore, we speculated that low USP25 expression is associated with impaired antitumor immunity in CD8^+^ T cells through the induction of MDSC accumulation (Fig. 3E). Furthermore, gene module analysis with the TIMER database revealed that USP25 expression was directly positively correlated with the infiltration of CD8^+^ T cells in HNSCC patients (Fig. 3F), but was significantly negatively correlated with the infiltration of MDSCs (Fig. 3G). Consistent with our findings from the scRNA-seq analyses and TIMER database, mIHC staining revealed that low USP25 expression was positively correlated with decreased CD8^+^ T-cell infiltration and elevated MDSC infiltration (Fig. 3H). These findings suggest a potential role for USP25 in regulating the immunosuppressive TIME of HNSCC.

### Overexpression of USP25 reduces the number of MDSCs but increases the infiltration of cytotoxic T cells

**Fig. 4:**
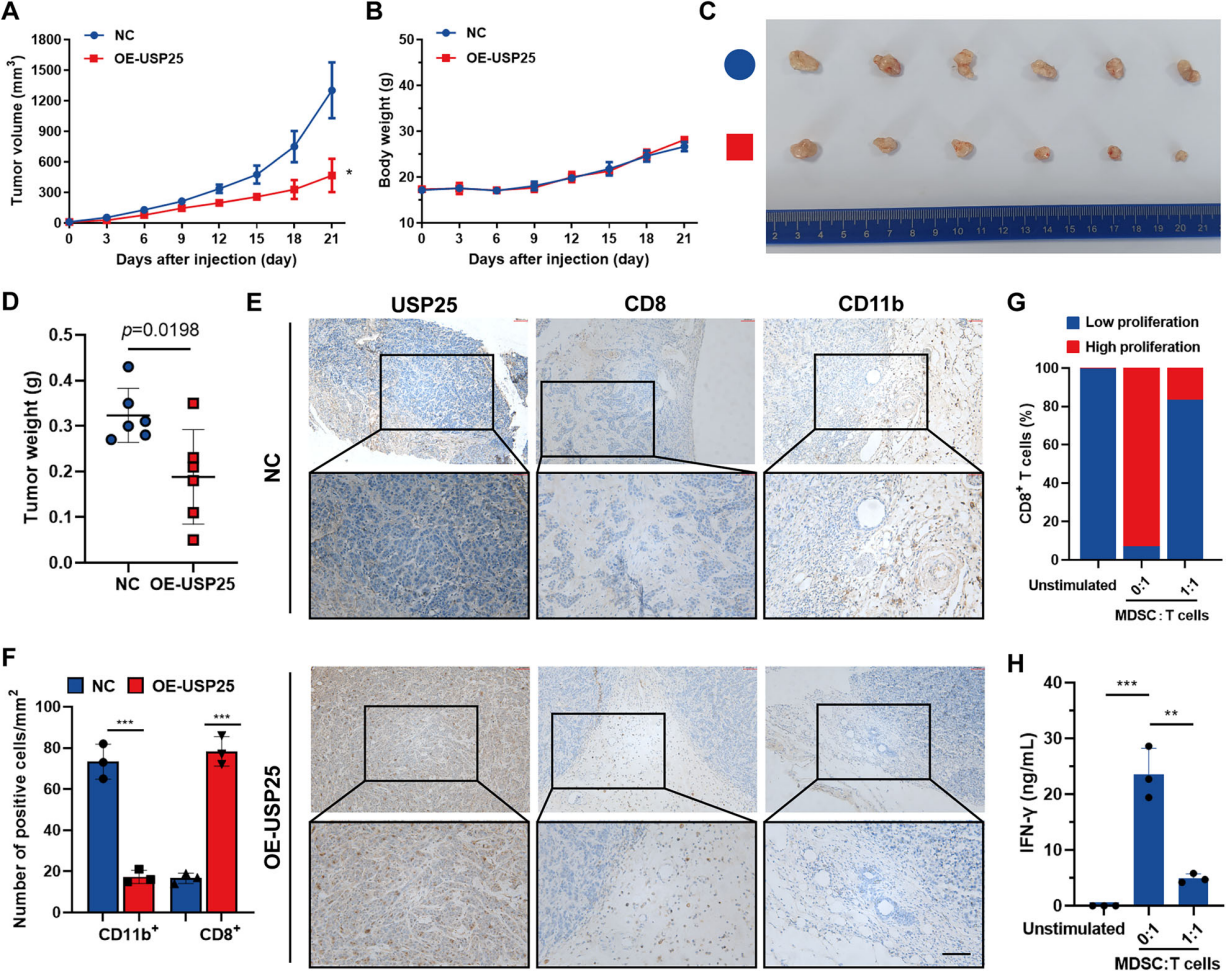
Overexpression of USP25 reduces the number of MDSCs but increases the degree of cytotoxic T-cell infiltration. **A** The tumor volume and body weight (**B**) were measured in the USP25 overexpression group and control group. **C** Representative images of HNSCC syngeneic tumors in the indicated groups. The size and weight of tumors significantly decreased in the USP25 overexpressing group (**D**). **E** IHC analysis for USP25, CD8^+^, and CD11b^+^ markers, indicating that MDSCs infiltrate USP25 overexpressing tumor tissues. **F** Statistical plot of mIHC staining. **G** CFSE assay for measuring the effect of MDSCs isolated from tumors on in vitro T-cell proliferation. **H** ELISA results showing changes in IFN-γ secretion by CD8^+^ T cells. Data represent the mean ± SD, *P < 0.05, **P < 0.01, ***P < 0.001.

To elucidate the contribution of USP25 to the progression of HNSCC tumors, we constructed a mouse syngeneic tumor model. Stable overexpression of USP25 resulted in a considerable decrease in tumor size (Fig. 4A), but the weight of the mice did not significantly change until the end of the experiment (Fig. 4B). After 21 days, the average weight of tumors in the USP25-overexpression group was significantly lower than that in the control group (Fig. 4C, D). IHC staining was used to detect the expression levels of CD8, CD11b, and USP25 (Fig. 4E), and the results verified a significant positive correlation between increased USP25 expression and reduced MDSC infiltration as well as increased T-cell infiltration in the tumors of tumor-bearing mice (Fig. 4F). Furthermore, the inhibitory effects of MDSCs isolated from tumors in the USP25 overexpression group on T-cell proliferation (Fig. 4G) and IFN-γ secretion (Fig. 4H) were weaker than those in the control group, indicating that USP25 overexpression reverses immunosuppressive status and induces an immune-activating antitumor microenvironment. Therefore, we concluded that low expression levels of USP25 resulted in an immunosuppressive TIME through aggravated MDSC infiltration and the exclusion of cytotoxic T cells in vivo.

### Upregulated USP25 expression inhibits MAPK pathway activation and IL-6 secretion, and attenuates MDSC migration

**Fig. 5:**
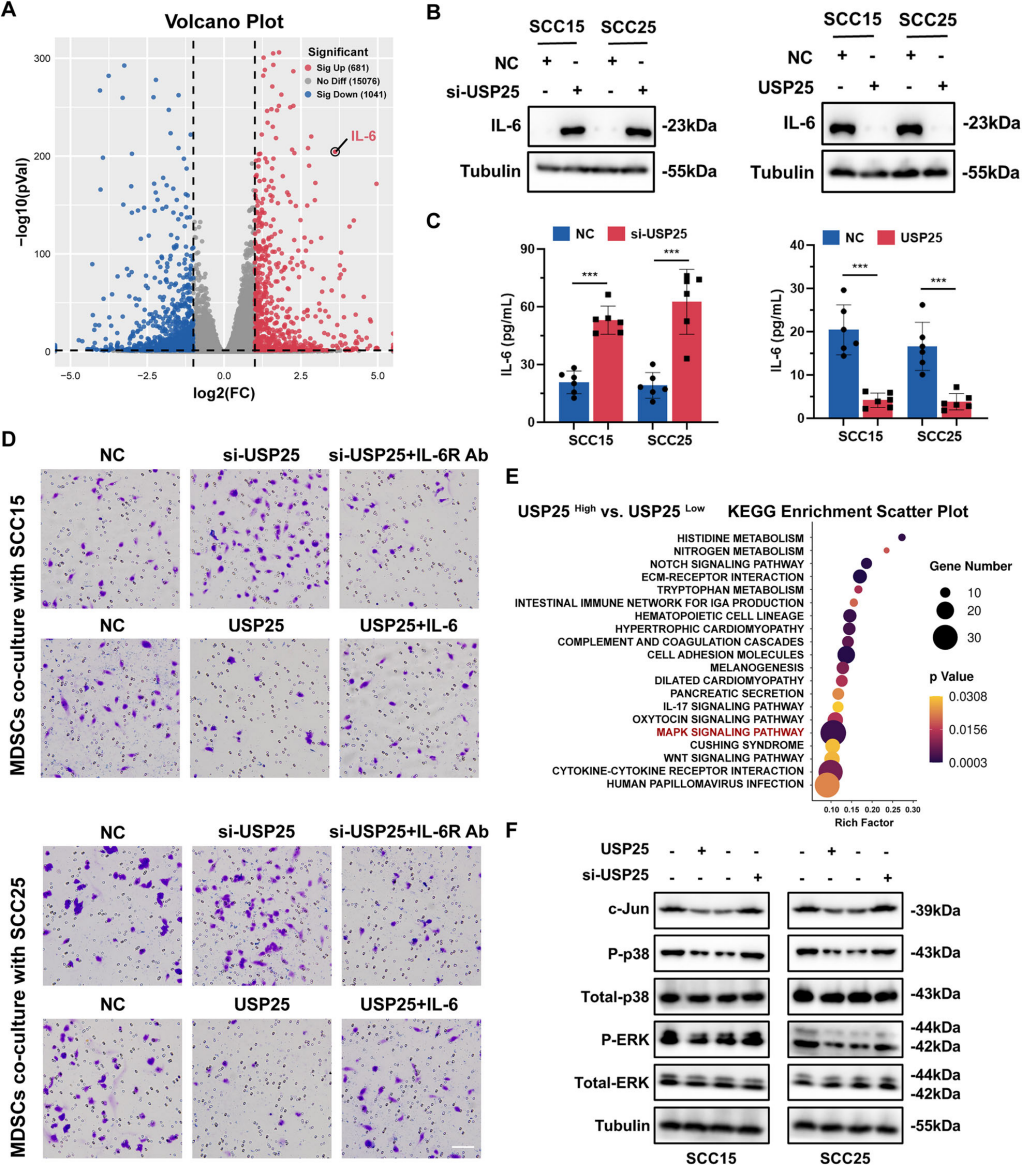
Upregulated USP25 inhibited MAPK pathway activation and IL-6 secretion and attenuated MDSC migration. **A** Volcano plot of the RNA-seq data (red and blue indicate significantly upregulated and downregulated genes, respectively). **B** Western blot analysis of IL-6 expression in HNSCC cells with USP25 downregulation or overexpression. **C** ELISA results showing the changes in IL-6 secretion in cell supernatants. **D** In vitro chemotaxis assay for MDSCs cocultured with SCC15 and SCC25 cells. Scale bar, 100 μm. **E** KEGG enrichment analysis revealed that MAPK signaling was significantly enriched. **F** The abundance of c-Jun, P-p38, p38, P-ERK, and ERK was measured by western blotting. Data represent the mean ± SD, *P < 0.05, **P < 0.01, ***P < 0.001.

To identify potential soluble factors secreted by HNSCC cells that aggravate MDSC infiltration in the TIME, we conducted RNA-seq on USP25 overexpressing cells and control cells to screen for differentially expressed genes. Among all the genes encoding soluble factors that exhibited differential expression in the groups (Table S2), IL-6 was the most dramatically differentially expressed gene encoding cytokines and chemokines on the basis of its highest fold change and lowest *P* value (Fig. 5A). IL-6 influences tumor progression through chemotactic MDSCs in the TIME [25]. Furthermore, we detected the expression of IL-6 in the SCC15 and SCC25 cell lines. We silenced USP25 expression in HNSCC cells using small interfering RNA (siRNA) transfection (Fig. S1E). USP25 overexpression dramatically decreased the mRNA (Fig. S1F, G) and protein expression of IL-6, and USP25 knockdown reversed these changes (Fig. 5B). Consistently, the ELISA results confirmed the changes in IL-6 secretion levels in cell supernatants following USP25 overexpression or downregulation (Fig. 5C). To assess the effect of USP25 levels on MDSC recruitment, we performed a chemotaxis assay involving the coculture of either HNSCC cells or MDSCs in vitro. Transwell coculture results revealed that MDSCs cocultured with tumor cells with decreased USP25 expression displayed increased chemotactic activity in vitro, and this effect was abrogated upon treatment with an anti-IL-6 receptor antibody (IL-6R Ab, Fig. 5D). The opposite trend was observed in MDSCs cocultured with USP25 overexpressing cancer cells, and the administration of IL-6 reversed this trend (Fig. 5D). To elucidate the pathways modulated by USP25, we evaluated how gene expression differed between the USP25 high and low expression groups using high throughput RNA-seq. We focused on the enriched pathways related to IL-6 synthesis and secretion and found that the MAPK signaling pathway was among the most prominently activated signaling pathways (Fig. 5E). Previous studies have demonstrated that the activation of MAPK signaling leads to the production of cytokines, such as IL-6. Notably, our previous studies confirmed that tumor-derived IL-6 significantly induced tumor growth and metastasis by promoting MDSC recruitment [26–28]. Consistent with these findings, western blotting confirmed that USP25 inhibited the activation of MAPK signaling and the expression of AP-1, a transcription factor of IL-6, in HNSCC cell lines (Fig. 5F). Collectively, these results indicate that upregulated USP25 expression inhibited MAPK pathway activation and IL-6 secretion, ultimately attenuating MDSC migration.

### USP25 binds to TAB2 and inhibits TAB2-mediated MAPK signaling transduction

**Fig. 6:**
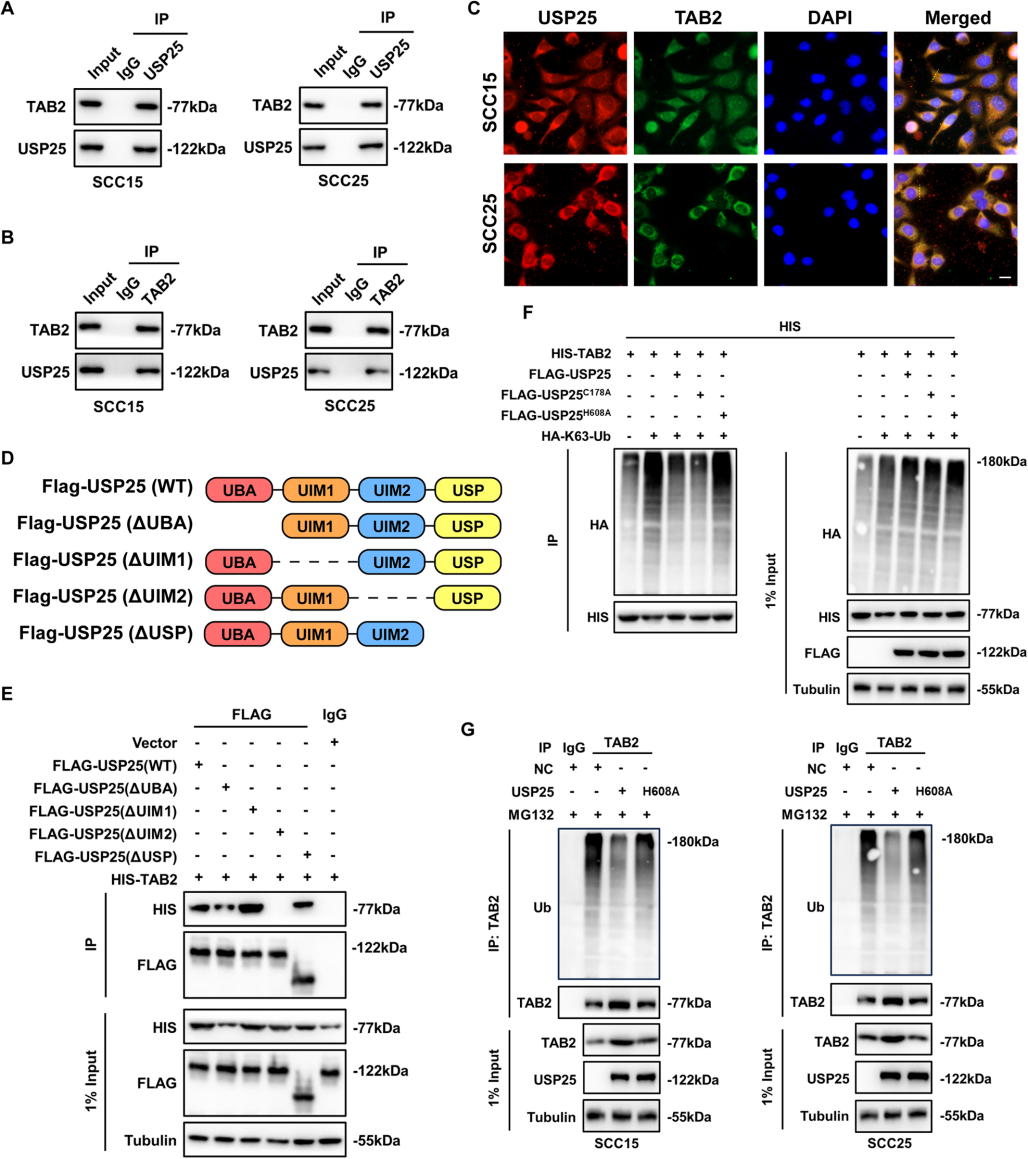
USP25 binds to TAB2 and inhibits TAB2-mediated MAPK signaling transduction. **A** Proteins were immunoprecipitated from cell lysates with anti-USP25 antibody or anti-TAB2 antibody (**B**) and then analyzed by immunoblotting. **C** Immunofluorescence of USP25 (red) and TAB2 (green) in HNSCC cells. Scale bar, 20 µm. **D** Schematic domain structure of USP25 and different mutants. **E** IP and immunoblot analysis of HEK293T cells transfected with the indicated plasmids. **F** HEK293T cells were transfected with the indicated plasmids, and proteins were immunoprecipitated with anti-HIS antibody and analyzed by immunoblotting. **G** The polyubiquitination level of endogenous TAB2 in SCC15 and SCC25 cells stably expressing USP25, catalytically inactive mutant of USP25 (H608A), was measured by an in vitro deubiquitination assay. Data represent the mean ± SD, *P < 0.05, **P < 0.01, ***P < 0.001.

To investigate the mechanism through which USP25 regulates the activation of the MAPK pathway, we immunoprecipitated USP25 and analyzed its interaction with common upstream regulators of signaling pathways. On the basis of results of a small-scale screening, TAB2 was identified as an interacting protein of USP25 (Fig. 6A, B), and USP25 did not interact with other upstream signaling molecules, including TAK1 and TAB3 (Fig. S1H). Immunofluorescence colocalization assays demonstrated that USP25 directly binds to TAB2 (Figs. 6C and S1I, J). Structurally, USP25 comprises a UBA domain, two UIM domains, and a USP domain. To investigate the domain-specific contributions to the binding interaction, a series of Flag-tagged plasmids lacking different domains were generated for the Co-IP assay (Fig. 6D). Our results demonstrated that the UIM2 domain of USP25 facilitated its physical association with TAB2 (Fig. 6E). K63 polyubiquitination of TAB2 is essential for the phosphorylation of TAK1, which subsequently activates downstream MAPK signaling, and previous studies have shown that the C178 and H608 residues in the USP domain are involved in the DUB activity of USP25 [28]. As illustrated in Fig. 6F, the K63 ubiquitination level of TAB2 was significantly attenuated by both wild-type USP25 and the C178A mutant but not by the H608A mutant, indicating that the H608 residue is required for the DUB activity of USP25 toward TAB2 (Fig. 6F). Through an in vitro deubiquitination assay, we confirmed that the K63 ubiquitination level of TAB2 was strongly elevated in HNSCC cells stably transfected with USP25 (Fig. 6G). This evidence illustrates that USP25 binds to TAB2 and inhibits MAPK signaling transduction through the K63-mediated deubiquitination of TAB2.

### USP25 overexpression enhances sensitivity to anti-PD-1 immunotherapy in vivo

**Fig. 7:**
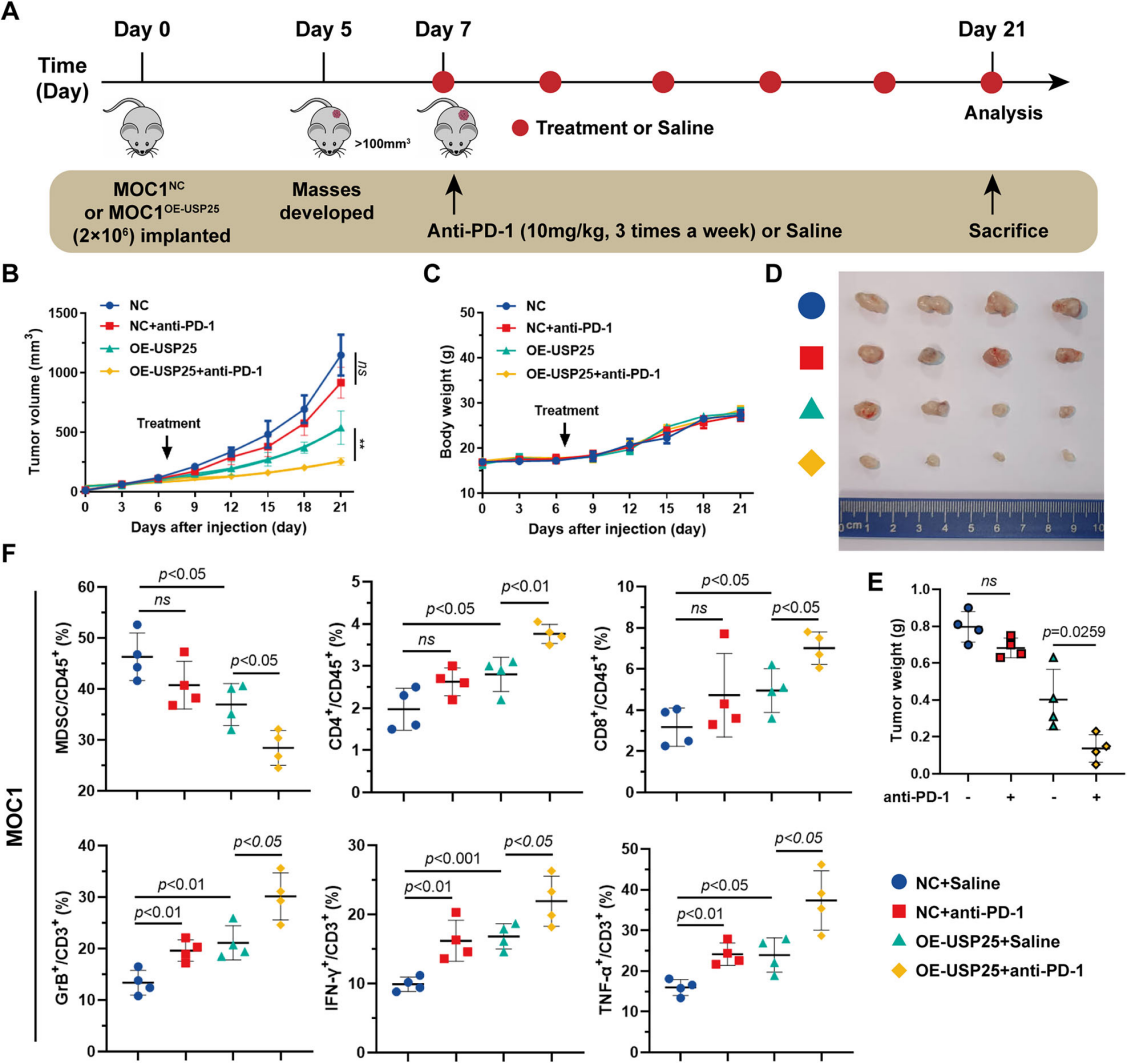
USP25 overexpression enhances sensitivity to anti-PD-1 immunotherapy in vivo. **A** MOC1 syngeneic tumors were established in immunocompetent C57 mice, and anti-PD-1 treatment was administered at the indicated times and doses. **B** Growth curve of subcutaneous tumors. **C** Graph of the change in weight of the mice vs. days of treatment. **D** Representative images and weights (**E**) of the tumors. **F** Top: FC data showing the proportions of MDSCs, CD4^+^ T cells, and CD8^+^ T cells. Lower: FC assessment of functional CD8^+^ T cells in tumors. Data represent the mean ± SD, *P < 0.05, **P < 0.01, ***P < 0.001.

Considering that reduced infiltration of MDSCs has been reported to correlate with increased sensitivity to immunotherapy efficacy in various cancer types [11, 14], we sought to test whether overexpression of USP25 augments anti-PD-1 therapy efficacy in HNSCC. To assess the effect of USP25 and the therapeutic efficacy of anti-PD-1 antibodies on HNSCC in vivo, we developed a mouse model in which MOC1^NC^ or MOC1^OE-USP25^ cells were implanted. When the MOC1 syngeneic tumors reached a volume of 50 ~ 100 mm^3^, we treated the mice with either saline or anti-PD-1 (10 mg/kg) treatment (Fig. 7A). The tumor volume was recorded every 3 days and our findings indicated that the tumors in the USP25 overexpression group were significantly smaller than those in the control group (Fig. 7B). Notably, no significant difference in mouse weight was observed among the treatment groups, suggesting limited toxicity (Fig. 7C). The results revealed that overexpression of USP25 sensitized the HNSCC tumors to anti-PD-1 treatment and resulted in lower tumor weights, whereas the tumors in the control group did not respond well to immunotherapy (Fig. 7D, E). Tumors characterized by more T-cell infiltration are referred to as hot tumors and are more sensitive to immunotherapy, resulting in better immunotherapy effects [14]. FC analysis further revealed that the combination of USP25 overexpression and anti-PD-1 therapy markedly reduced the accumulation of MDSCs, but strongly increased the number of tumor-infiltrating functional CD8^+^ T cells, including Granzyme B^+^ T cells, IFN-γ^+^ T cells, and TNF-α^+^ T cells (Fig. 7F). Therefore, overexpression of USP25 not only attenuates tumor growth but also potentiates the therapeutic efficacy of ICB in HNSCC by suppressing the recruitment of MDSCs and improving the functionality of CD8^+^ T cells.

## Discussion

USP25, a DUB, has emerged as a key regulator in multiple cancers and is frequently downregulated in HNSCC. However, its role in antitumor immunity is largely unknown. The TIME arises from the complex interrelationship between antitumor immunity and the tumor itself, and plays a crucial role in tumor progression and prognosis [29]. In the present study, our data demonstrated for the first time that low USP25 expression in HNSCC drives immunosuppression. Single-cell profiling revealed that USP25 depletion significantly increased the recruitment of MDSCs, which in turn contributed to the inhibition of T-cell infiltration and function in HNSCC, thereby reinforcing the immunosuppressive TIME. This phenomenon is consistently corroborated across clinical specimens, cellular assays, and murine models. Collectively, our results provide innovative perspectives on potential immunotherapeutic interventions for HNSCC patients.

Ubiquitination and deubiquitination systems are pivotal in regulating signaling pathways that govern various biological and pathological processes, such as cancer [30]. To date, numerous DUBs have been identified as key participants in multiple cellular processes within cancer cells, such as cell growth [31], differentiation [32], transcriptional regulation [33, 34], and oncogenesis [35]. Given the critical roles DUBs play in cancer progression, we conducted a bioinformatics analysis to screen 10 DUBs in HNSCC. Among these, USP25 has emerged as a candidate protein known for its ability to remove ubiquitin chains from target proteins, thereby facilitating their degradation [22, 36]. Our findings revealed that USP25 expression lower in tumor tissues than in adjacent normal tissues and that this reduced expression is significantly associated with poor prognosis in HNSCC. However, in vitro cellular assays indicated that USP25 does not directly affect the malignant biological behaviors of HNSCC cells. Consequently, we redirected our attention to inhibitory immune cells, such as TAMs and MDSCs, within the TIME. A series of in vivo and in vitro experiments demonstrated that USP25 plays a role in the recruitment of MDSCs in HNSCC. MDSCs, a heterogeneous group of immature myeloid cells, are crucial contributors to the immunosuppressive TIME [37]. In vitro migration assays further confirmed that USP25 is essential for MDSC chemotaxis. Collectively, these findings suggest that USP25 inhibits HNSCC progression by alleviating MDSC-mediated immunosuppression.

MDSCs exhibit potent immunosuppressive capabilities and are recognized as pivotal regulators of immune evasion, primarily by inhibiting the proliferation and function of key effector cells, including CD8^+^ T cells, CD4^+^ T cells, and natural killer (NK) cells [38]. Notably, MDSCs can be recruited into the TIME, where they play a critical role in tumor immunotherapy [39]. Previous studies have demonstrated that tumor cells secrete cytokines, including IL-6 and CXCL3, among others, to facilitate the recruitment of MDSCs into the TIME [40, 41]. However, the underlying mechanisms remain poorly understood. In this study, we elucidated the molecular basis of USP25-associated MDSC accumulation in HNSCC. Gene set enrichment analysis revealed that MAPK signaling was significantly activated in the USP25 low expression group. The MAPK pathway, which is frequently activated in tumor cells, is known to regulate the expression of proinflammatory cytokines [42]. We further identified the c-JUN subunit of AP-1 as a direct target influenced by MAPK activation. Through cytokine profiling, we observed that IL-6 expression and secretion were suppressed by the overexpression of USP25 in HNSCC. Upon ligand-receptor binding, tumor-derived IL-6 plays a crucial role in promoting MDSC chemotaxis within the TIME by activating the JAK/STAT3 and NF-κB signaling pathways [43, 44]. Consistent with these findings, we revealed that USP25 depletion enhances MDSC migration via the release of IL-6, an effect that was abolished by IL-6 receptor blockade. These findings collectively suggest that USP25-mediated MAPK signaling drives MDSC infiltration through IL-6 secretion in HNSCC.

Mechanistically, we discovered that high expression of USP25 inhibits the activation of the MAPK signaling pathway in HNSCC, which is consistent with findings from previous studies [28, 45]. Zhong et al. reported that USP25 negatively regulates MAPK activation by removing K63-linked ubiquitination from TRAF5 and TRAF6 [45]. Additionally, Li et al. demonstrated that USP25 suppresses TLR-4-induced activation of the NF-κB and MAPK signaling pathways in microglia [28]. Although the functional role of USP25 in HNSCC has not been previously explored, our study identified TAB2 as a key target of USP25. TAB2, an adaptor protein, activates TAK1 by binding to Lys63-linked polyubiquitin chains. Our experimental results revealed that USP25 interacts with and deubiquitinates TAB2 at K63, thereby inhibiting the MAPK pathway. Notably, we further elucidated that USP25 interacts with TAB2 specifically through its UIM2 domain. These findings underscore the role of USP25 in modulating IL-6 expression via TAB2, ultimately influencing the immunosuppressive TIME in HNSCC.

In the clinic, immunotherapeutic strategies have proven to be effective against multiple tumors, and researchers are increasingly focusing on immunotherapy for patients with HNSCC. To date, only a subset of HNSCC patients respond to immunotherapies [6]. Clinical trials have shown that the infiltration of multiple immunocytes into the TIME affects the efficacy of immunotherapy. This can be partially attributed to the infiltration of MDSCs and the formation of an immunosuppressive TIME [14]. Therefore, reversing the suppressive TIME is a promising strategy for improving immunotherapy outcomes [11]. Our study indicated that USP25 overexpression could systematically restore the immunocompetent TIME by reducing MDSC infiltration and recruiting cytotoxic T cells in HNSCC and could enhance the tumor response to ICB therapy. Similarly, multiple studies have confirmed that MDSC depletion significantly enhances immunotherapy efficacy [46]. Intratumoral lymphocyte infiltration is clearly essential for effective ICB therapy, which offers insights into the future development of HNSCC treatment strategies. Our findings highlight a strategy that involves converting “cold” tumors into “hot” tumors, which might provide a promising treatment option for HNSCC patients.

**Fig. 8:**
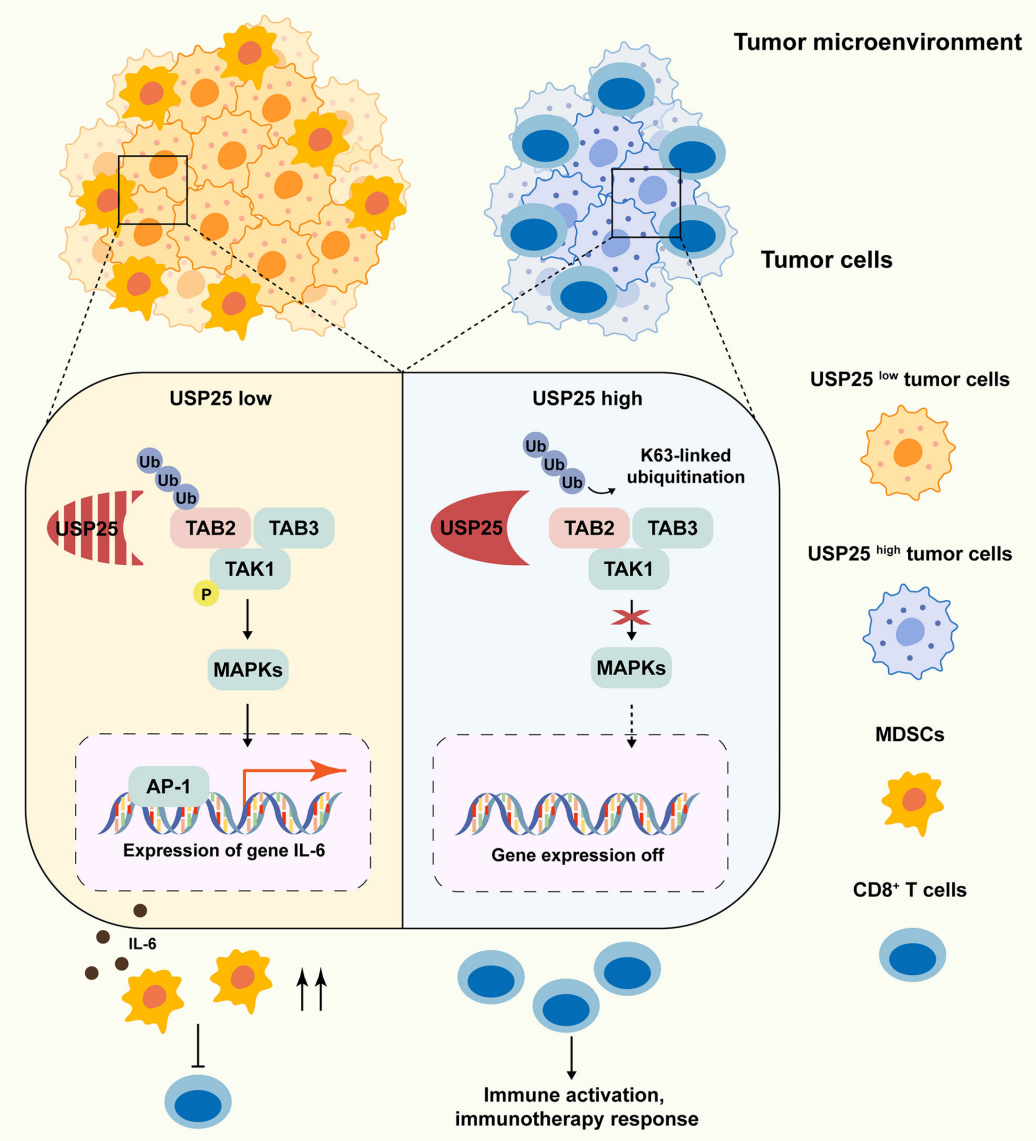
Schematic illustration of the role of USP25 in regulating the immunosuppressive TIME in HNSCC. USP25 inhibits the activation of the MAPK signaling pathway by binding to TAB2 and removing K63-specific polyubiquitin chains, thereby inhibiting MDSC accumulation and hence bolstering T-cell function in HNSCC.

In conclusion, our findings demonstrated a novel role of USP25 in HNSCC through its role in suppressing the ubiquitination of TAB2. This effectively suppressed MAPK signaling activation, thereby inhibiting the recruitment of MDSCs, conferring a response to IBC therapy, and attenuating the malignant progression of HNSCC (Fig. 8). We anticipate that this work contributes to a deeper understanding of the regulatory network governing the TIME, offering promising insights for the treatment of HNSCC patients.

## Materials and methods

### Patient information

For this study, 12 paired fresh HNSCC tissues and NATs, and 12 fresh HNSCC tissues with or without lymphatic metastasis (with lymphatic metastasis, 6 patients; without lymphatic metastasis, 6 patients) were obtained from patients underwent Lumpectomy at the department of Thyroid Head and Neck Surgery of the Affiliated Cancer Hospital of Zhengzhou University. Additionally, we obtained 56 cases of HNSCC tissue specimens from a tissue microarray (TMA). Experiment protocols have been approved by the Ethics Committees of Zhengzhou University and written informed consents were obtained from the patients.

### Bioinformatics analysis

We analyzed 95 potential DUBs using RNA-sequencing data in raw form regarding HNSCC were derived online from The Cancer Genome Atlas (TCGA) database and Gene Expression Omnibus (GEO) database (GSE33205 and GSE37991). The levels of USP25 in normal tissues, dysplasia lesions, and carcinoma was enrolled from GEO database (GSE30784). The single-cell RNA-sequencing data was acquired from the GEO database (GSE181919). The online software GEPIA (gepia2.cancer-pku.cn) was used to conduct the TCGA analysis, and GEO2R was used to identify gene expression in GEO database. Analysis and visualization of the survival was performed with the Kaplan–Meier Plotter website tool (https://www.kmplot.com). Analysis and visualization of the data were conducted with R software (version 4.0.3).

### Cell lines and cell culture

HNSCC cell lines SCC15 and SCC25 were obtained from the American Type Culture Collection (ATCC, USA). The MOC1 cell line was purchased from the KeraFAST (Boston, USA). The human HNSCC cell lines SCC15 and SCC25 were cultured in DMEM/F-12 supplemented with 10% FBS. The mouse oral cavity squamous cell carcinoma cell line MOC1 was cultured in IMDM MOC line media. Cells were cultured in conditions that imitated humidified environments, with a temperature of 37 °C and a CO_2_ concentration of 5%. Furthermore, authentication through short tandem repeat genotyping was conducted to verify the cells before the experiments.

### Antibodies and reagents

The antibodies and reagents employed in this study were listed in the Table S1 for various techniques, including immunohistochemistry (IHC), multiplex immunohistochemistry (mIHC), immunoblotting (IB), immunofluorescence (IF), Co-immunoprecipitation (Co-IP), and flow cytometry (FC) assays.

### Immunohistochemistry staining and scoring analyses

The immunohistochemistry (IHC) staining experiment was conducted utilizing a detection kit (Zhongshan Jinqiao, SP-9000, Beijing, China) in accordance with the protocol. In short, the paraffin sections or TMA needed to undergo deparaffinization and rehydration. H_2_O_2_ was used to prevent interference from endogenous peroxidase, and the citric acid buffer was used for antigen retrieval by microwave. After antigen retrieval, the sections were blocked with goat serum, and then incubated with primary antibodies over night at 4 °C (Table S1). On the second day, the sections were treated with biotinylated secondary antibodies utilizing the immunoperoxidase method and subsequently incubated with diaminobenzidine and hematoxylin for color development. Finally, it was dehydrated, transparent, and mounted with neutral gum. Mounted specimens were visualized and analyzed by ImageScope software. The staining distribution scores were determined according to the intensity × proportion of stained tumour cells, and the median value was chosen as the cutoff.

### Fluorescent multiplex immunohistochemistry

Fluorescent multiplex immunohistochemistry (mIHC) was performed with Opal Manual kit (Akoya Biosciences) according to the manufacturer’s protocol. Briefly, mIHC technique is based on a series of staining rounds in which the secondary antibody is labeled with a fluorescent molecule coupled to the Tyramide Signal Amplification System (TSA). Heat-treated stripping of the tissues between the staining rounds removes the primary and secondary antibodies but not the TSA-conjugated fluorescence molecules. The slides were scanned for whole-slide imaging using the Mantra quantitative pathology imaging system.

### Transfection and transduction

Small interfering RNAs (siRNAs) targeting human USP25 and negative control were obtained from GENCEFE Biotech (Wuxi, China). HA-tagged USP25a (Tet-On) were constructed by using pCMV-HA as a backbone. Transfection was performed using *Lipofecta*-mine 3000 (Invitrogen) according to the standard protocols, lentivirus package and transduction was conducted as previously described [47].

### Protein extraction and immunoblotting

Cells were collected and lysed using RIPA lysis buffer (Beyotime). The quantified proteins were resolved by SDS-PAGE gels and transferred onto polyvinylidene fluoride membranes (Merck Millipore), then the membranes were blocked with 5% non-fat milk for 1 h at room temperature and incubated with the corresponding antibodies overnight at 4 °C. After HRP-conjugated secondary antibody incubation was applied, an enhanced chemiluminescence reagent (MilliporeSigma) was used for imaging with a chemiluminescence imaging system.

### Transwell assay

The in vitro invasion capacity of HNSCC cells was examined using transwell chamber (Corning). SCC15 cells or SCC25 cells (1 × 10^5^ cells/well) were added to the upper compartment of the chamber, and treatment medium containing 20% fetal bovine serum (FBS) was added to the lower chamber. After, cells invading to the lower side of the membrane were immobilized with methanol and stained with 0.1% crystal violet, then counted using a microscope.

### Cells proliferation CCK8 assay

Cells’ proliferation was assessed by using CCK8 assay kit according to the manufacturer’s instructions (Servicebio, Wuhan, China). The OD at 450 nm was tested using a microplate reader (Multiskan FC, Thermo Fisher Scientific Inc).

### Immunofluorescence

SCC15 and SCC25 cells were seeded on 18 mm cover glasses until they adhered to the surface. After methanol immobilization, cells were fixed and permeabilized with 0.2% Triton X-100. The samples were blocked with 5% BSA, followed by an overnight incubation with primary antibodies USP25 and TAB2. For immunofluorescence detection, cells were incubated with a fluorescence-conjugated secondary antibody. The images were captured using LSM 880 laser scanning confocal microscope (Zeiss, Oberkochen, Germany).

### Co-immunoprecipitation assay

Co-immunoprecipitation assays were performed as described [48]. The HNSCC cell lysates were incubated with the anti-USP25 antibodies or IgG and washed by the lysis buffer. The beads-bound proteins were analyzed by immunoblotting using indicated antibodies.

### Enzyme-linked immunosorbent assay (ELISA)

The levels of IL-6 and IFN-γ content in the supernatants or tissue samples were assessed using commercially available ELISA kits according to manufacturer’s instructions.

### In vitro ubiquitination assays

To investigate endogenous TAB2 ubiquitination, HNSCC cells were incubated with MG132 (10 μM) for 12 h and lysed with RIPA buffer. Anti-TAB2 antibodies was applied to immunoprecipitating proteins and separate ubiquitinated TAB2. The endogenous ubiquitin chains on TAB2 were immunoblotted with anti-ubiquitin antibody.

### Animal experiments

All animal protocols were in accordance with the requirements of the Animal Care and Use Committee of Zhengzhou University. 2 × 10^6^ MOC1 cells were implanted subcutaneously into 5-week-old female C57 mice until xenografts established (>100 mm^3^). The mice were administered with saline, or Pembrolizumab (anti-PD-1, 10 mg/kg) every 3 days, respectively. Tumor volume and body weight were examined every 3 days. The tumor volume (mm^3^) was calculated with the following formula: tumor volume = (longest axis × shortest axis^2^)/2. After 21 days, the animals were terminated, and the tumors were harvested for further studies.

### Statistical analysis

Statistical analyses were performed using GraphPad Prism software (version 8.0). The comparisons between two groups were performed by two-tailed Student’s *t* tests. Multiple-group comparisons were performed by one-way or two-way ANOVA. The correlation between genes was performed using Pearson correlation test. Survival curves were performed using the log-rank (Mantel–Cox) test. Data were presented as mean ± standard deviation. Statistical significance was set at *P* < 0.05, and denoted by asterisks throughout the figures as follows: n.s. (not significant), *(*P* < 0.05), **(*P* < 0.01), ***(*P* < 0.001).

## Supplementary information

Supplementary files

Original western blots

## Data Availability

The datasets used and/or analyzed during the current study are available from the corresponding author on reasonable request.
