# Supplementary material for: USP25 attenuates the immunosuppressive tumor microenvironment via the deubiquitination of TAB2 in head and neck squamous cell carcinoma
Source: Cell Death Discov. 2025 Dec 1;12:27. doi: 10.1038/s41420-025-02883-1 (PMC12811241; doi:10.1038/s41420-025-02883-1)
Supplement: Supplementary file 1 — Supplementary files [file 41420_2025_2883_MOESM1_ESM.docx]

**Supplementary Files**

**Supplementary Figures**


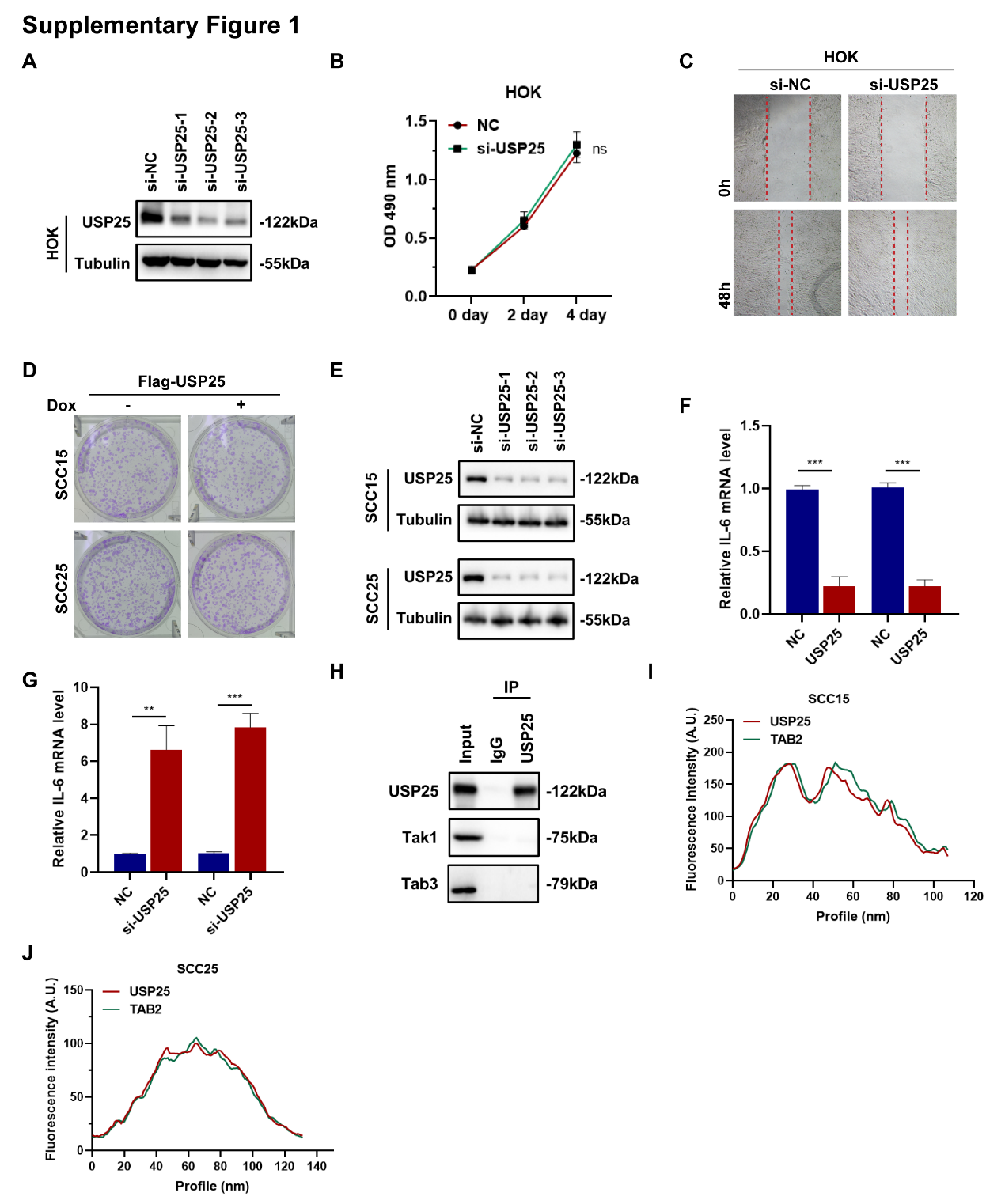


**Figure S1.** The expression of USP25. (A) The protein expressions of USP25 were measured in HOK cells transfected with si-USP25 or si-NC. (B) CCK8 assays for HOK cell line. (C) Wound-healing assays for HOK cells. (D) Clonogenicity assays for SCC15 and SCC25 cells with USP25 overexpression. (E) The protein expressions of USP25 were measured in SCC15 and SCC25 cells transfected with si-USP25 or si-NC. (F-G) The mRNA level of IL-6 was analyzed by qRT-PCR assay. (H) Proteins were immunoprecipitated from cell lysates with anti-USP25 antibody or IgG, and subsequently analyzed by western blot with indicated antibodies. (I-J) The Fluorescence intensities of USP25 and TAB2 along with the yellow lines. Data represent mean ± SD, *P < 0.05, **P < 0.01, ***P < 0.001.

**Supplementary Tables**

**Table S1.** Key resources table.

| **Reagent/Resource** | **Dilution** | **Applications** | **Source** | **Identifier** |
| --- | --- | --- | --- | --- |
| **Antibodies** | | | | |
| USP25 | 1:200; 1:200; 1:1000 | IHC; mIHC; IB; IP | Proteintech | #12199-1-AP |
| CD8 | 1:5000 | IHC; mIHC | Proteintech | #66868-1-Ig |
| TAB2 | 1:1000; 1: 200 | IB; IF; IP | Proteintech | #14410-1-AP |
| Ubiquitin | 1:1000 | IB | Proteintech | #10201-2-AP |
| USP25 | 1:200 | IF | Abcam | #ab246948 |
| HA | 1:1000 | IB | Abcam | #ab9110 |
| FLAG | 1:500; 1:500 | IHC; mIHC | Abcam | #ab205606 |
| HIS | 1:1000 | IB | Abcam | #ab18184 |
| Pan-CK | 1:250 | mIHC | Abcam | #ab7753 |
| Tubulin | 1:5000 | IB | Abcam | #ab176560 |
| IL-6 | 1:1000 | IB | Abcam | #ab9324 |
| CD11b | 1:200; 1:200 | IHC; mIHC | Abcam | #ab133357 |
| CD8 | 1:200 | IHC | Servicebio | #GB15068 |
| CD11b | 1:200 | IHC | Servicebio | #GB15058 |
| c-JUN | 1:1000 | IB | Servicebio | #GB111604 |
| Phospho-p38 | 1:1000 | IB | Servicebio | #GB113380 |
| Total-p38 | 1:1000 | IB | Servicebio | #GB114685 |
| Phospho-ERK | 1:1000 | IB | Servicebio | #GB11004 |
| Total-ERK | 1:1000 | IB | Servicebio | #GB11560 |
| PerCP Rat Anti-Mouse CD45 |  | FC | BD Biosciences | Cat#557235 |
| FITC Rat Anti-Mouse CD11b |  | FC | BD Biosciences | Cat#557396 |
| PE Rat Anti-Mouse Ly6G and Ly6C |  | FC | BD Biosciences | Cat#553128 |
| FITC Rat Anti-Mouse CD3 |  | FC | BD Biosciences | Cat#555274 |
| PE Rat Anti-Mouse CD8 |  | FC | BD Biosciences | Cat#550798 |
| **Chemicals** |  |  |  |  |
| IL-6 |  |  | MedChemExpress | #HY-P7044 |
| IL-6R Ab |  |  | Selleck | #A2011 |
| Pembrolizumab |  |  | Selleck | #A2005 |
| **ELISA kits** |  |  |  |  |
| IL-6 |  | ELISA | Abcam | #ab178013 |
| IFN-γ |  | ELISA | Bioss | Bsk12001 |
| IB: immunoblotting; IHC: immunohistochemistry; mIHC: multiplex immunohistochemistry; IF: immunofluorescence; IP: immunoprecipitation; ELISA: Enzyme-linked immunosorbent assay | | | | |

**Table S2.** List of the genes encoding soluble factors.

| **Up** | **Down** |
| --- | --- |
| IL1A | IL12A |
| IL1RN | IL15 |
| IL1B | IL16 |
| IL6 | IL23A |
| IL10 | IL32 |
| IL11 | IL34 |
| IL17B | CCL11 |
| IL17D | CCL26 |
| IL18 | CXCL1 |
| IL24 | CXCL6 |
| IL33 | CXCL10 |
| CCL2 | CXCR4 |
| CCL5 | CSF1 |
| CCL7 | TGFB1 |
| CCL16 | TGFB2 |
| CCL24 | TGFB3 |
| CCL20 | CLEC3B |
| CCL22 | ANG |
| CCL28 | COMP |
| CXCL2 | CGB2 |
| CXCL3 | COLEC10 |
| CXCL5 | FAM180A |
| CXCL8 | FAM3A |
| CXCL12 | FSTL3 |
| CXCL16 | HYAL3 |
| CX3CL1 | IGFBP4 |
| CSF2 | PRDX4 |
| CXF3 | PLA2G15 |
| CXCR2 | SHBGTSLP |
| HMGB1 |  |
| HMGB2 |  |
| HMGA2 |  |
| HDGF |  |
| PDGFA |  |
| VEGFA |  |
| FGF5 |  |
