# Supplementary material for: USP25 attenuates the immunosuppressive tumor microenvironment via the deubiquitination of TAB2 in head and neck squamous cell carcinoma
Source: Cell Death Discov. 2025 Dec 1;12:27. doi: 10.1038/s41420-025-02883-1 (PMC12811241; doi:10.1038/s41420-025-02883-1)

Figure 1K

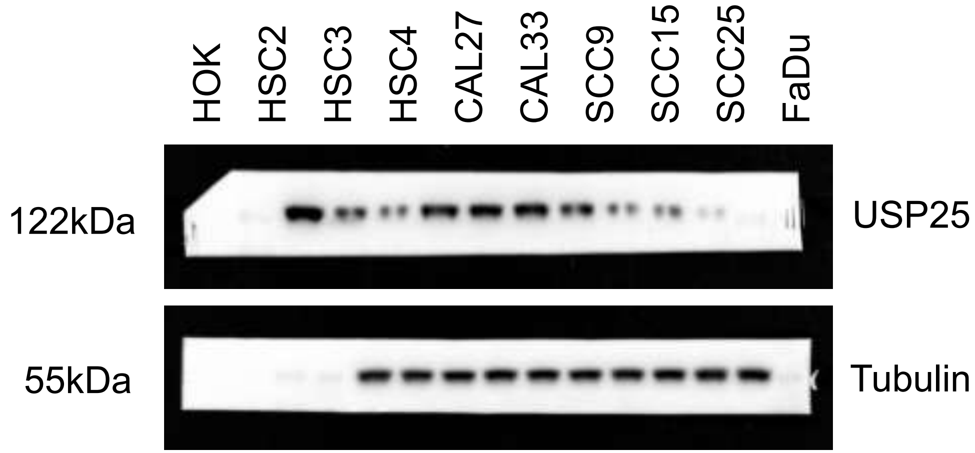

Figure 5B

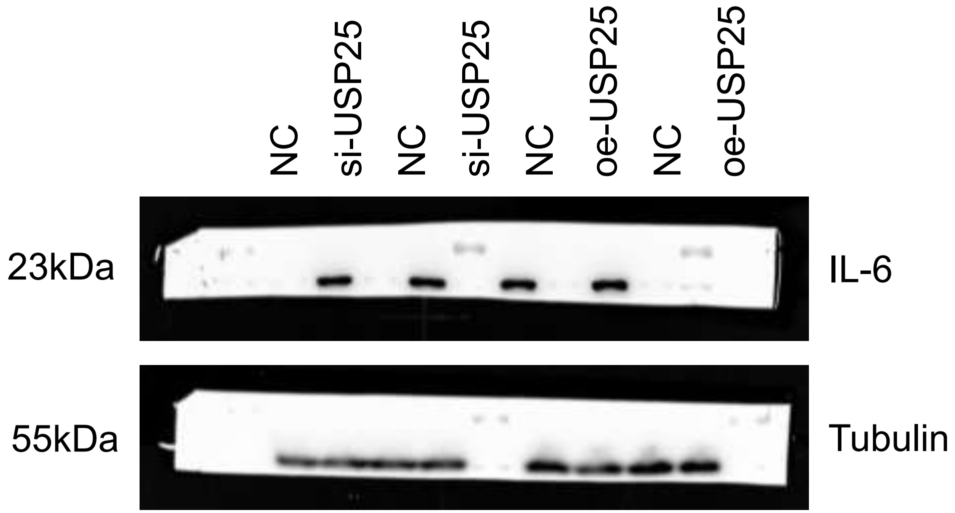

Figure 2G

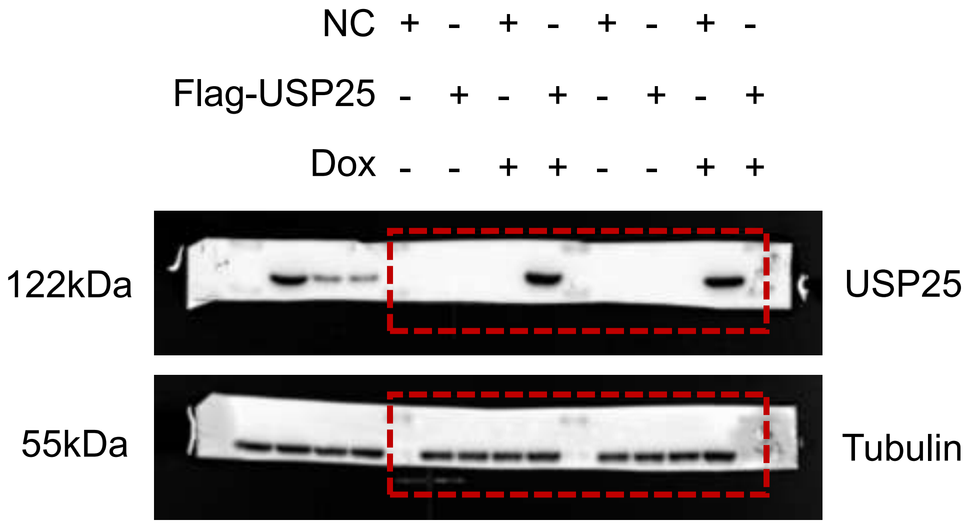

Figure 5F

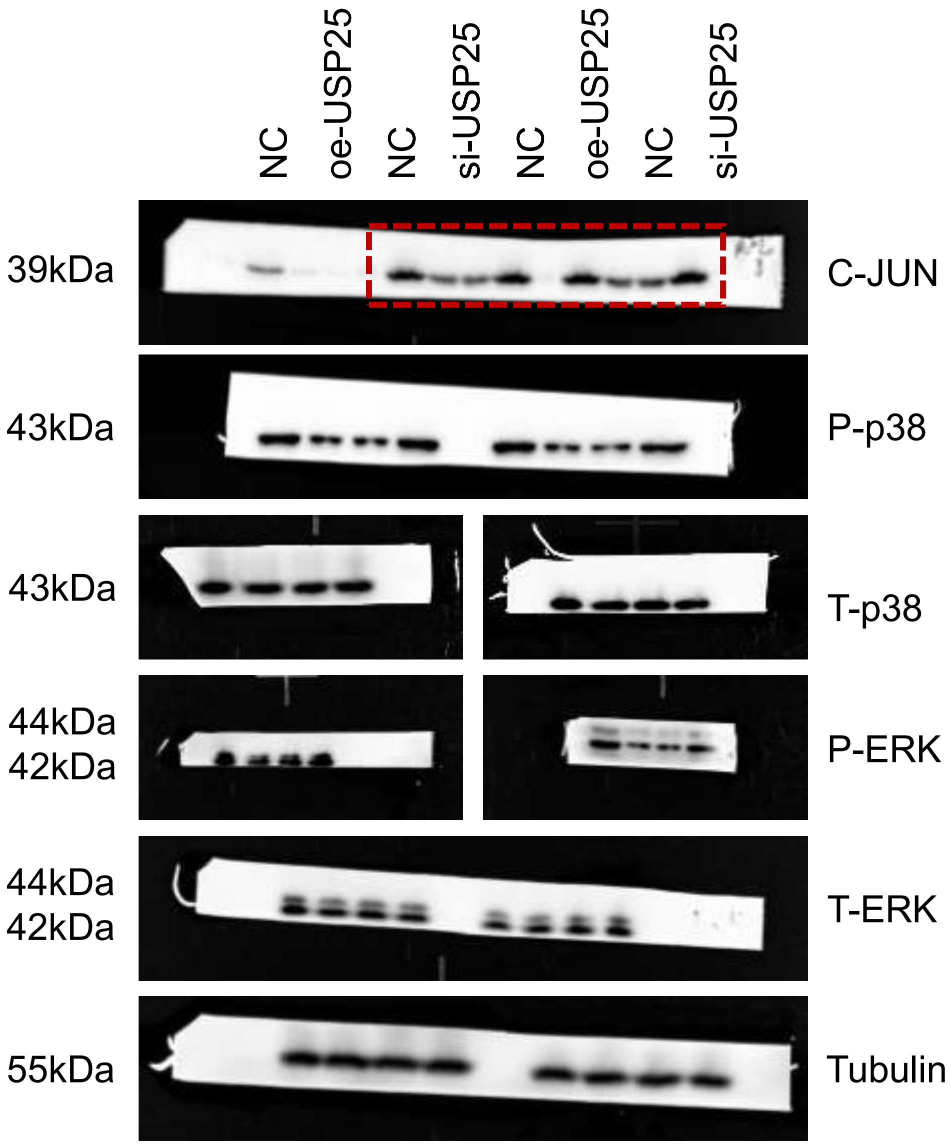

Figure 6A

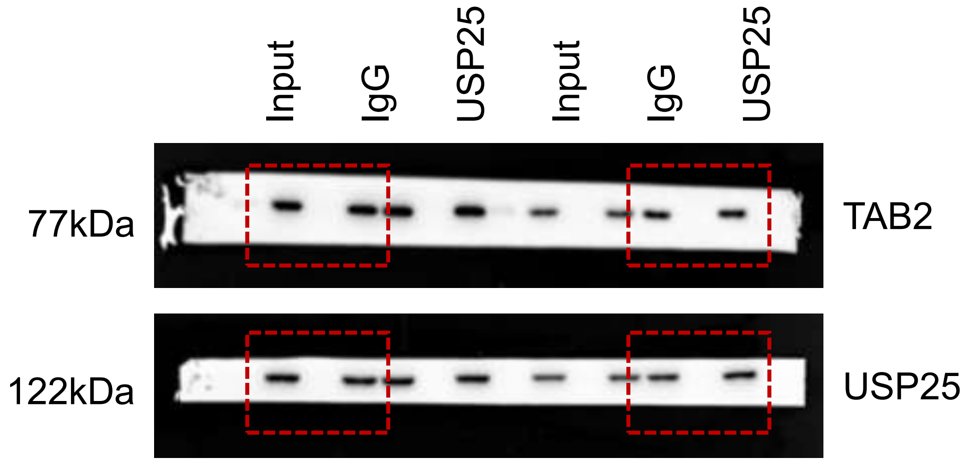

Figure 6B

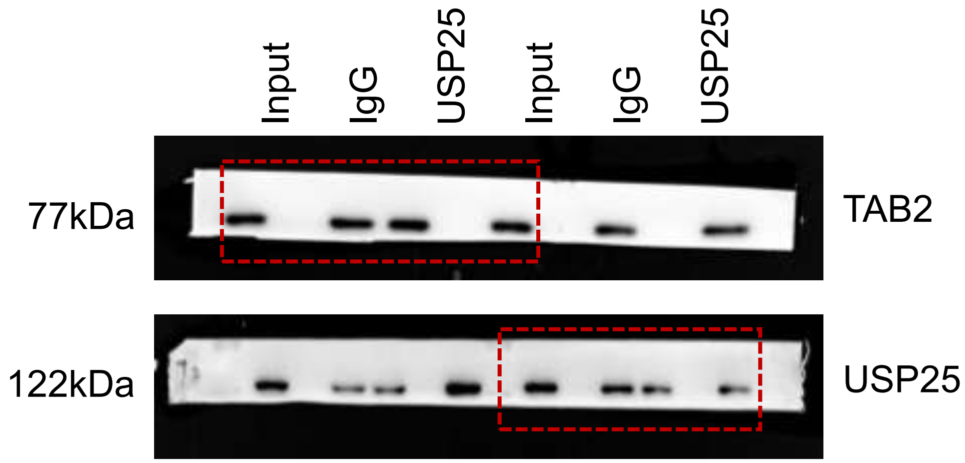

Figure 6E

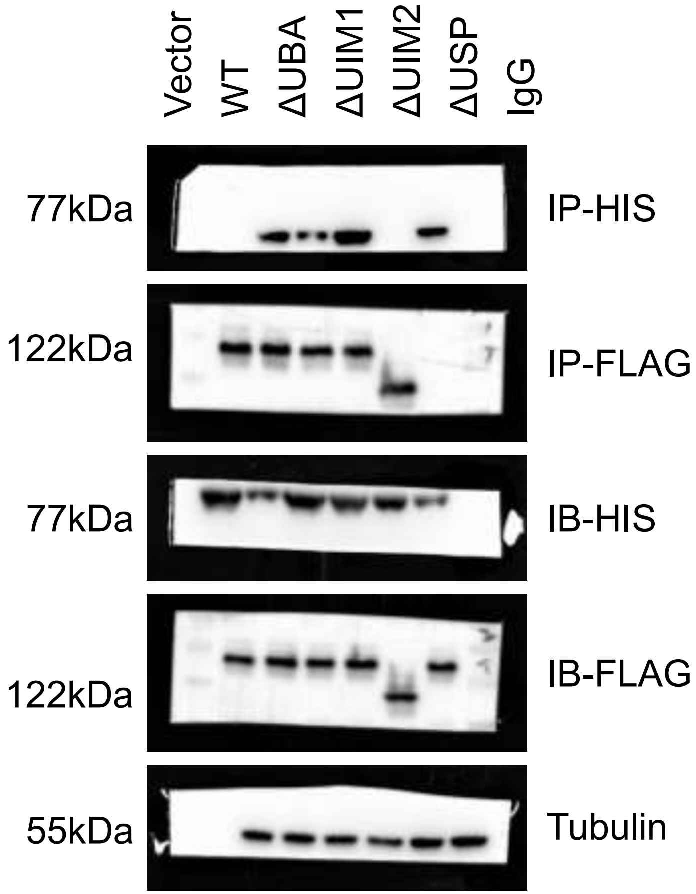

Figure 6F

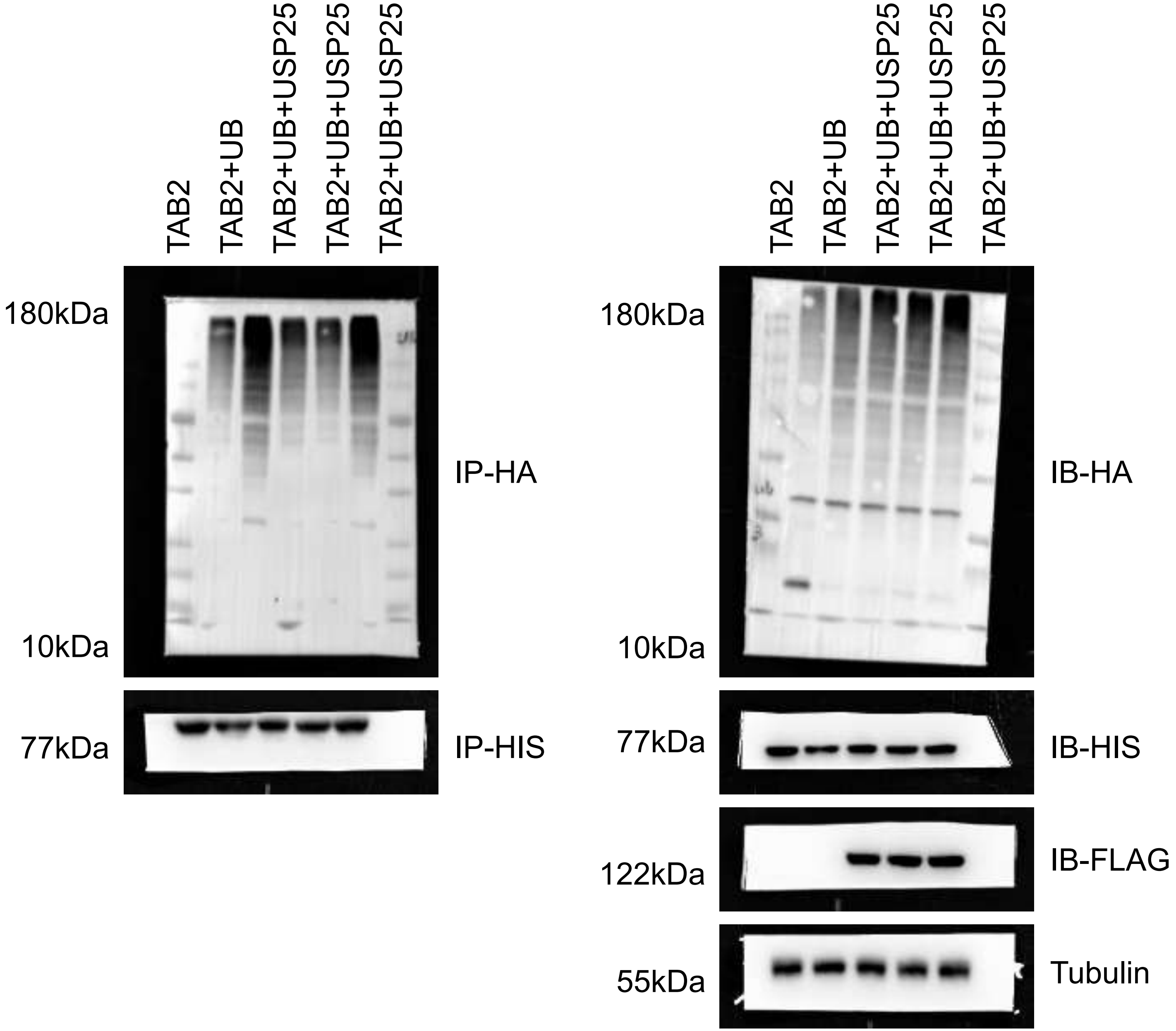

Figure 6G

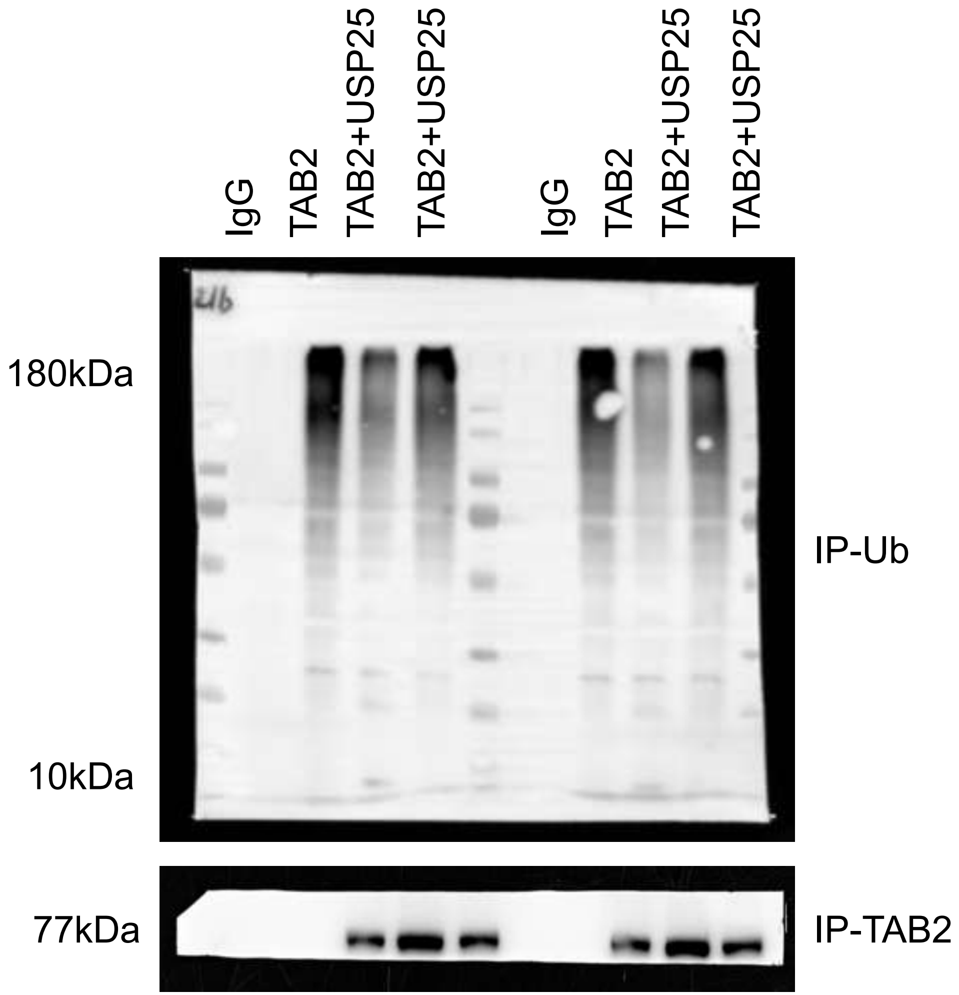
IgG
TAB2
TAB2+USP25
TAB2+USP25
IgG
TAB2
TAB2+USP25
TAB2+USP25
180kDa
10kDa
IP-Ub
77kDa
IP-TAB2

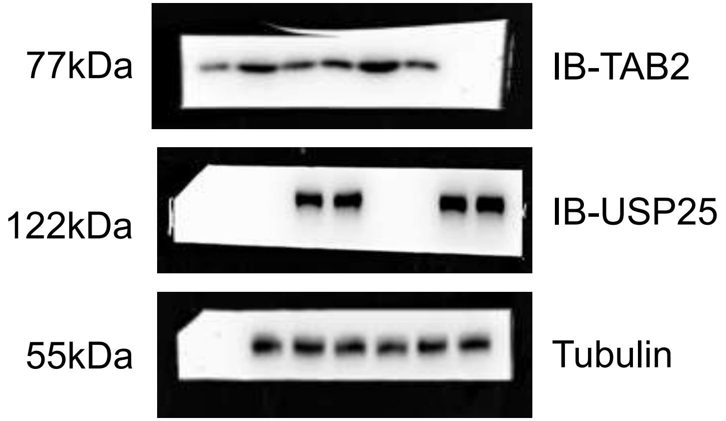
77kDa
IB-TAB2
122kDa
IB-USP25
55kDa
Tubulin

Figure S1B

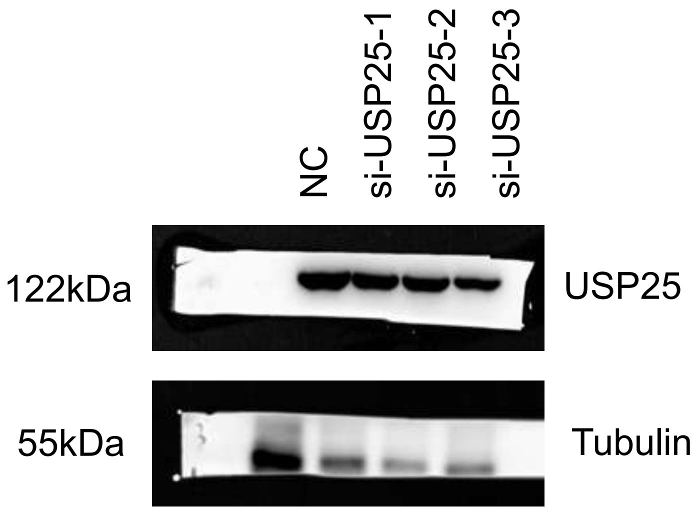

Figure S1E

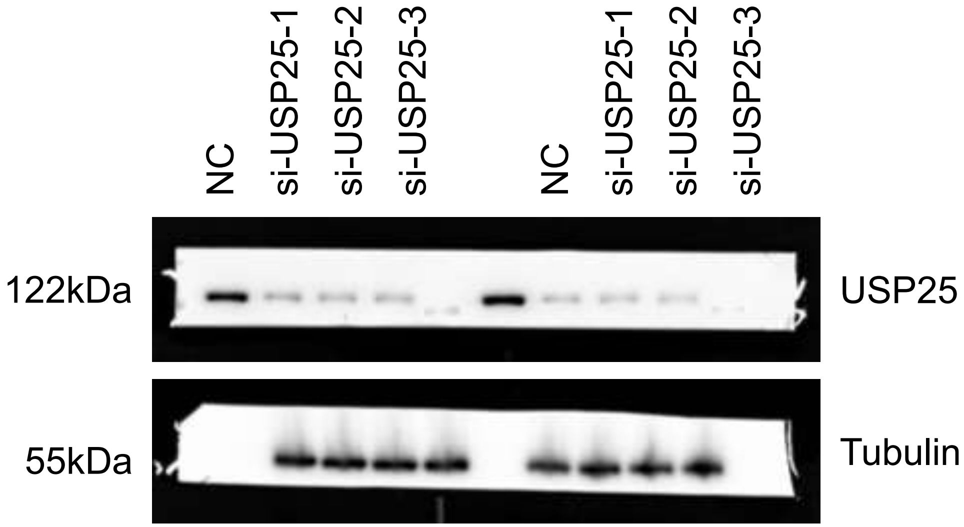

Figure S1H

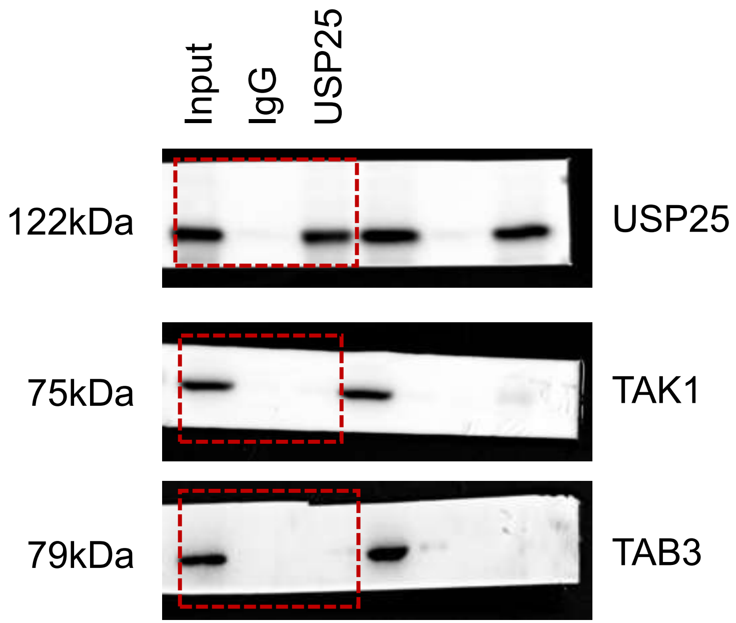

Supplement: Supplementary file 2 — Original western blots [file 41420_2025_2883_MOESM2_ESM.pdf]
